# Investigating associations of delay discounting with brain structure, working memory, and episodic memory

**DOI:** 10.1093/cercor/bhac164

**Published:** 2022-04-30

**Authors:** Benjamín Garzón, Zeb Kurth-Nelson, Lars Bäckman, Lars Nyberg, Marc Guitart-Masip

**Affiliations:** Aging Research Center, Department of Neurobiology, Care Sciences and Society, Karolinska Institutet, Tomtebodavägen 18A, 17 165, Stockholm, Sweden; Max Planck UCL Centre for Computational Psychiatry and Ageing Research, University College London, 10-12 Russell Square, WC1B 5EH, London, United Kingdom; Aging Research Center, Department of Neurobiology, Care Sciences and Society, Karolinska Institutet, Tomtebodavägen 18A, 17 165, Stockholm, Sweden; Department of Radiation Sciences, Umeå University, 3A, 2tr, Norrlands universitetssjukhus, 901 87, Umeå, Sweden; Umeå Center for Functional Brain Imaging, Umeå University, Linnaeus väg 7, 907 36, Umeå, Sweden; Department of Integrative Medical Biology, Umeå University, H, Biologihuset, 901 87, Umeå, Sweden; Aging Research Center, Department of Neurobiology, Care Sciences and Society, Karolinska Institutet, Tomtebodavägen 18A, 17 165, Stockholm, Sweden; Max Planck UCL Centre for Computational Psychiatry and Ageing Research, University College London, 10-12 Russell Square, WC1B 5EH, London, United Kingdom

**Keywords:** delay discounting, intertemporal choices, structural networks, magnetic resonance imaging, brain structure

## Abstract

**Introduction:**

Delay discounting (DD), the preference for smaller and sooner rewards over larger and later ones, is an important behavioural phenomenon for daily functioning of increasing interest within psychopathology. The neurobiological mechanisms behind DD are not well understood and the literature on structural correlates of DD shows inconsistencies.

**Methods:**

Here we leveraged a large openly available dataset (*n* = 1196) to investigate associations with memory performance and gray and white matter correlates of DD using linked independent component analysis.

**Results:**

Greater DD was related to smaller anterior temporal gray matter volume. Associations of DD with total cortical volume, subcortical volumes, markers of white matter microscopic organization, working memory, and episodic memory scores were not significant after controlling for education and income.

**Conclusion:**

Effects of size comparable to the one we identified would be unlikely to be replicated with sample sizes common in many previous studies in this domain, which may explain the incongruities in the literature. The paucity and small size of the effects detected in our data underscore the importance of using large samples together with methods that accommodate their statistical structure and appropriate control for confounders, as well as the need to devise paradigms with improved task parameter reliability in studies relating brain structure and cognitive abilities with DD.

## Introduction

When confronted with the dilemma of choosing between a reward that will materialize immediately and a larger reward that will be deferred, we often choose the former. This behavior, termed temporal or delay discounting (DD), can be observed in humans and animals and is interpreted from an economics standpoint as a decrease in the subjective value of items when a subject needs to wait for a delay to obtain them (Samuelson 1937; Story et al. 2016). People discount both primary rewards, like food, drugs, or sex, and monetary rewards (Mcclure et al. 2007; Odum 2011).

Choices involving rewards awarded at different delays (intertemporal choices) are omnipresent and can have significant consequences in our lives. A certain amount of discounting can be an adaptive feature in many situations, for instance those in which the delivery of future rewards is highly uncertain (Story et al. 2016). Nevertheless, there are remarkable interindividual differences in how people discount future rewards, and maladaptive intertemporal choices are frequently observed in mental disorders such as addiction (Mackillop et al. 2011; Amlung et al. 2017), schizophrenia (Ahn et al. 2011), and depression (Takahashi et al. 2008; Pulcu et al. 2014). Within the field of psychiatry, there is great interest in DD because of its potential to meet the Research Domain Criteria (Cuthbert and Insel 2013) as a behavioral dimension of psychopathology that can reveal underlying neurobiological characteristics (Lempert et al. 2018).

A common way to measure DD is to present subjects with binary choices between a smaller reward given immediately and a larger reward given in the future (“choose between 50 USD today or 100 USD in 6 months”) and fit a model that predicts their responses based on a hypothetical discount rate (Samuelson 1937; Mazur 1987). The level of discounting thus measured shows high test–retest reliability and can be regarded as a trait of the subject (Odum 2011; van den Bos et al. 2014).

Generally, studies investigating the link between structural brain differences and DD converge on a network of regions thought to support reward valuation, episodic prospection, and top–down control (Peters and Büchel 2011; Owens et al. 2017), but they have yielded partially conflicting results, possibly due to small sample sizes (Kable et al. 2015; Pehlivanova et al. 2018). Negative associations with discount rates have been reported with gray matter volume (GMV) or thickness of the lateral prefrontal cortex (PFC) (Bjork et al. 2009; Cho et al. 2013), superior frontal gyrus (Schwartz et al. 2010), medial PFC, anterior cingulate cortex (Bernhardt et al. 2014), and putamen (Dombrovski et al. 2012). On the other hand, greater DD has been related to a larger volume of the ventral striatum and posterior cingulate cortex (Schwartz et al. 2010) and increased local GMV in frontal pole and middle frontal gyrus (Wang et al. 2016), medial prefrontal and anterior cingulate regions (Cho et al. 2013), and caudate (Tschernegg et al. 2015); some of these positive associations contradict the negative ones mentioned above. The largest study to date in younger adults found greater discounting with smaller GMV in the middle temporal gyrus and entorhinal cortex (Owens et al. 2017).

The literature linking white matter differences to DD is even sparser and consists of studies with relatively small samples. DD has been related to variation in the superior (Mohammadi et al. 2016) and inferior longitudinal fasciculi (Olson et al. 2009; Herting et al. 2010; Mohammadi et al. 2016) and fronto-striatal tracts (Peper et al. 2013; Hänggi et al. 2016; Hampton et al. 2017). Olson et al. (2009) found in 79 adolescents an association with white matter regions including portions of the inferior and superior longitudinal fasciculi, anterior thalamic radiation, uncinate fasciculus, inferior fronto-occipital fasciculus, corticospinal tract, and splenium of the corpus callosum. Adolescent subjects with stronger connectivity of the pathway between striatum and right dorsolateral PFC tend to be less impatient, and the development of this tract has been linked to a decrease in discounting as teenagers grow older (van den Bos et al. 2015). The largest study in the field thus far (Han et al. 2018) found a negative association between temporal discounting and white matter integrity in bilateral frontal, fronto-striatal, and temporoparietal tracts in older persons.

How interindividual differences in the aforementioned neural correlates lead to differences in DD is still unknown. One of the candidate cognitive mechanisms that may underlie this relationship is working memory (WM), which has been associated negatively with DD (Shamosh et al. 2008; Ahn et al. 2011). WM training has been shown to decrease discounting in adults in treatment for stimulant addiction (Bickel et al. 2011). A meta-analysis of functional activations of WM and DD tasks (Wesley and Bickel 2014) pinpointed a unique region in the posterior portion of the left lateral PFC, where WM and DD functional activation maps overlap. The authors of that study hypothesized that this area is associated with integrating temporal information about the recent past and the foreseeable future into the ongoing executive processes involved in eliciting a decision. Further, choice difficulty when comparing immediate and delayed rewards modulates activity within WM-related PFC regions (Jimura et al. 2018). Another cognitive mechanism that has been related to DD is episodic memory (EM). Subjects tend to be more patient after retrieving positive, but not negative, autobiographical memories (Lempert et al. 2017). Episodic future thinking engages common regions with remembering past experiences (medial prefrontal regions, medial and lateral parietal cortex, lateral temporal cortex, and the medial temporal lobe) (Addis et al. 2007; Schacter et al. 2007; Schacter and Addis 2009) and also reduces DD (Peters and Büchel 2010; Daniel et al. 2015; Bromberg et al. 2017; Hu et al. 2017). The degree to which this modulation occurs is predicted by the functional coupling between hippocampus and anterior cingulate cortex (Peters and Büchel 2010) and also medial PFC (Benoit et al. 2011). From a computational perspective, it has been proposed that temporal discounting consists of a search process where the subjective value of future rewards depends on the ability to find representations of these rewards in memory, with rewards associated with events that are farther away in time being more difficult to find in a putative representational space, therefore being assigned a smaller subjective value (Kurth-Nelson et al. 2012). This search process relies thus on EM to store reward representations and WM to consider the costs and benefits of alternatives (Wesley and Bickel 2014).

In the present study, we analyzed behavioral and neuroimaging data from a large cohort of younger adults, the Human Connectome Project (HCP) dataset (Van Essen et al. 2013) (http://www.humanconnectome.org). Specifically, we investigated the volume of the cortex and of subcortical structures as well as a comprehensive set of covariance networks (i.e. networks of regions with strongly covarying values of structural measures) of gray and white matter structure. Greater DD was correlated with smaller GMV in anterior temporal cortex but, in spite of the large sample size, none of the associations with total cortical volume, subcortical volumes, or markers of microscopic organization of white matter were significant. Besides, we found no evidence of an association of either WM or EM with DD in the tasks of the HCP dataset.

## Materials and methods

### Participants

HCP participants were 1,196 younger adults (mean age = 28.9, SD = 3.7 years, 547 men and 646 women) eligible for magnetic resonance imaging and with no significant history of neuropsychiatric disorders, substance abuse, cardiovascular disease, or Mendelian genetic disease. All participants provided written informed consent (Van Essen et al. 2012). The number and demographic characteristics of the subjects with valid data for the different cognitive tasks and imaging modalities are shown in Table 1. In each analysis that follows we included all the subjects with complete data for the variables relevant to it. The dataset is described in more detail elsewhere (Van Essen et al. 2013).

**Table 1:** Demographic characteristics of the sample used in the study, and number of available observations for the different modalities.

Demographic information								
*N*	1,196							
Age mean (SD)	28.8 (3.7)							
Gender (Male/Female)	543/651							
Years of education	14.9 (1.8)							
Income $1,000–$9,999/year $10,000–$19,999/year $20,000–$29,999/year $30,000–$39,999/year $40,000–$49,999/year $50,000–$74,999/year $75,000–$99,999/year $100,000–$149,999/year	7.49% 8.58% 12.70% 11.43% 10.43% 21.03% 12.95% 15.39%							
Valid observations (only cases with valid DD data)								
HCP dataset								
	DD	LSWMT	2-back	PWMT	PSMT	T1-w	Subcortical segmentation	DWI
Number of subjects	1,018	1,018	923	1,018	1,017	946	946	905

DWI, diffusion-weighted imaging, concerning FA and MD; T1-w concerns cortical thickness and area, GMV, and subcortical volume data.

### D‌D task

**Fig. 1:**
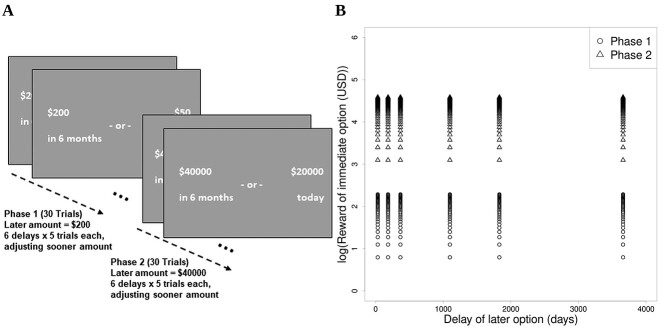
Intertemporal choice task. A) Intertemporal choice task: Subjects had to choose between a smaller immediate reward and a larger delayed reward over a number of trials. The DD task had 2 phases: In phase 1, the reward for the delayed option was 200 USD, while in phase 2 it was 40,000 USD, constant across trials. B) The plot shows delays for the later option and rewards for the sooner (immediate) option across trials. The scale of the *y*-axis is in days, but delays were presented to participants in more natural time units (days, months, years).

Participants were administered a computerized intertemporal choice task. Over a number of trials, they were presented binary choices between a smaller reward given immediately and a larger reward given in the future (Fig. 1A). Choices were hypothetical (i.e. subjects were not paid the amount they chose). We modeled participants’ subjective values of the rewards presented with a hyperbolic discounting function (Mazur 1987) with discount rate $k$. Larger $k$ values denote a greater discounting of future rewards and reflect more impatient behavior. We used all the trials when fitting the model, as discount rates estimated separately for small and large magnitude trials were highly correlated, and a parameter recovery analysis showed that fitting them separately led to considerably worse recovery. See the Supplementary Material for further details about the task and model, as well as auxiliary analyses.

### WM and EM scores

Participants completed the NIH Toolbox List Sorting Working Memory Test (LSWMT) (Tulsky et al. 2014) and a 2-back WM task (Barch et al. 2013). In addition, verbal EM was assessed with the Penn Word Memory Test (PWMT) (Gur et al. 2001) and nonverbal EM with the NIH Toolbox Picture Sequence Memory Test (PSMT) (Tulsky et al. 2014). The scores of all the tasks were Box-Cox transformed to render them normally distributed and subsequently standardized (transformation exponent: LSWMT: 0.90; 2-back: 3.88; PWMT: 4.46; PSMT: 1.31). More details about the tasks are provided in the Supplementary Material.

### Magnetic resonance imaging

#### Data acquisition and processing

Structural T1-weighted and diffusion-weighted imaging (DWI) scans were acquired (see Supplementary Material for scanning parameters and data processing particulars). Surface-based maps of cortical thickness and area, total cortical volumes, and volumes of subcortical structures (accumbens, amygdala, caudate, hippocampus, pallidum, putamen, and thalamus) for each hemisphere were generated with FreeSurfer (Fischl and Dale 2000). We also processed the T1-weighted images with a voxel-based morphometry (Ashburner and Friston 2000) pipeline (FSL-VBM) (Douaud et al. 2007) to produce individual GMV maps (modulated gray matter probability images). The analysis of GMV maps complements the previous one on structural volumes, as it does not assume that effects will occur in regions conforming to prespecified anatomical structures. Further details about the different gray matter measures and rationale for their inclusion are provided in the Supplementary Material.

From the DWI data, we computed fractional anisotropy (FA) and mean diffusivity (MD) maps, sensitive to the microscopic structure of white matter through its constraints on the diffusion of water protons in brain tissue (Le Bihan et al. 2001). The FA and MD maps were submitted to tract-based spatial statistics (TBSS) (Smith et al. 2006) to obtain individual maps restricted to the centers of all fiber bundles generally common to the study participants (tract skeletons, see Supplementary Material for further details).

### Statistical analyses

#### *Relationship between DD and WM/*EM

We tested for associations between DD and WM/EM, adjusting for linear and quadratic age terms, sex, years of education completed, and income level (the 2 latter according to the Semi-Structured Assessment for the Genetics of Alcoholism, Bucholz et al. (1994), included in the HCP battery). Five cases in which education and income had not been recorded were imputed based on sex and age using the mice package for R.

#### Independent component analysis

We decomposed the gray-matter structure modalities (cortical thickness and area maps and the GMV images) jointly into 50 components using linked independent component analysis (linked-ICA), a data-driven method that breaks down the data into a set of components of across-subject variability (covariance networks), which have been shown to have biological plausibility (Groves et al. 2011, 2012; Douaud et al. 2014; see Supplementary Material for further details). Each component is associated with a spatial map and a set of scores, one for each subject, together with relative weights corresponding to each of the input modalities (thickness, area, and GMV). A subject’s score for a certain component reflects the signed magnitude of the contribution of the component in the subject’s map. In a similar manner, we also decomposed the white-matter structure modalities (FA and MD) into 50 components. In order to verify the robustness of the results to varying the number of components, these analyses were repeated decomposing the data in 100 components instead of 50.

#### Relationship between structural predictors and DD

We tested for associations between the variables of interest (subcortical volumes, gray matter and white matter components, EM, WM) and DD with linear models and nonparametric permutation tests as implemented in FSL’s PALM software version 1.11 (Winkler et al. 2014; Winkler, Webster, et al. 2016b) running on MATLAB R2017b. We used 10,000 permutations, modeling the tail of the permutation distribution of *P*-values with a Pareto distribution (Winkler, Ridgway, et al. 2016a), and family-wise error rate (FWER) control for multiple tests using the distribution of the maximum statistic (Westfall and Young 1993). The HCP dataset contains subjects who were sampled along their siblings (mostly their twins), which means that the measurements cannot be regarded as independent. To test for associations between variables of interest while accounting for the family structure in the dataset, we used multilevel block permutation testing (Winkler et al. 2015). These associations were corrected for linear and quadratic age terms, sex, education, income, and the cognitive function composite score of the NIH Toolbox (“CogTotalComp_Unadj”), a measure of general intelligence. We report 2-tailed *P*-values for the associations tested.

#### Vertex- and voxel-wise analyses

For comparison, we also performed separate univariate vertex-wise (cortical thickness, surface area) and voxel-wise (GMV, FA, MD) analyses to test for associations with DD. These analyses were performed using nonparametric permutation tests as implemented in FSL’s PALM software, with 2,000 permutations, threshold-free cluster enhancement (Smith and Nichols 2009). As in the previous analyses, we modeled the tail of the permutation distribution of *P*-values with a Pareto distribution, applied FWER control for multiple tests using the distribution of the maximum statistic, and account for family structure with multilevel block permutation testing. Like above, these analyses were controlled for linear and quadratic age terms, sex, education, income, and general intelligence.

## Results

We excluded subjects with incomplete responses in the DD tasks (3), who chose either always immediate or delayed responses (9), or for whom the posterior probability of the pseudo-*R*^2^ statistic (Camerer and Ho 1999) being above zero was below 0.95, i.e. subjects for which the model fitted responses better than a chance model (166). These criteria led to the exclusion of 178 subjects from the HCP sample. Table 1 displays demographic information about the samples and number of valid observations for the different cognitive tasks and imaging modalities that were analyzed in relation to DD. The subjective value of rewards was on average 79% of their nominal value after a month and 23% after a year. Gender differences in DD were not significant (male (*n* = 473) > female (*n* = 545): *t* = 0.27, *P* = 0.80).

### *Relationship between DD and WM/*EM

After adjusting for linear and quadratic age terms, sex, education, and income, discount rate was not significantly associated with LSWMT scores (*r* = −0.022, *P* = 0.502, *n* = 1018) nor with 2-back scores (*r* = −0.007, *P* = 0.842, *n* = 923). The associations with verbal (PWMT; *r* = −0.045, *P* = 0.144, n = 1018) and nonverbal EM scores (PSMT; *r* = 0.032, *P* = 0.320, *n* = 1017) were not significant either.

### Relationship between DD and gray matter

After correcting for linear and quadratic age terms, sex, and total intracranial volume, discount rate was not associated with total cortical volume or the volume of any of the subcortical structures investigated. Table 2 displays the statistics for these associations.

**Table 2:** Associations between cortical and subcortical volumes and log-discount rates (*N* = 945).

Cortical volume	*r* = −0.032, *P*_uncorr_ = 0.062, *P*_corr_ = 0.524		
		Left Hemisphere	Right Hemisphere
Subcortical volumes	Accumbens	*r* = 0.013, *P*_uncorr_ = 0.673, *P*_corr_ = 1.000	*r* = −0.022, *P*_uncorr_ = 0.438, *P*_corr_ = 0.998
	Amygdala	*r* = 0.002, *P*_uncorr_ = 0.944, *P*_corr_ = 1.000	*r* = 0.031, *P*_uncorr_ = 0.226, *P*_corr_ = 0.928
	Caudate	*r* = −0.022, *P*_uncorr_ = 0.462, *P*_corr_ = 0.998	*r* = −0.025, *P*_uncorr_ = 0.399, *P*_corr_ = 0.989
	Hippocampus	*r* = −0.004, *P*_uncorr_ = 0.885, *P*_corr_ = 1.000	*r* = 0.014, *P*_uncorr_ = 0.588, *P*_corr_ = 1.000
	Pallidum	*r* = 0.014, *P*_uncorr_ = 0.641, *P*_corr_ = 1.000	*r* = 0.019, *P*_uncorr_ = 0.496, *P*_corr_ = 0.999
	Putamen	*r* = 0.010, *P*_uncorr_ = 0.712, *P*_corr_ = 1.000	*r* = 0.018, *P*_uncorr_ = 0.522, *P*_corr_ = 1.000
	Thalamus	*r* = 0.028, *P*_uncorr_ = 0.258, *P*_corr_ = 0.961	*r* = 0.043, *P*_uncorr_ = 0.084, *P*_corr_ = 0.606

Associations were estimated correcting for linear and quadratic terms of age, sex, education, income, and total intracranial volume. None of the associations were significant.

**Fig. 2:**
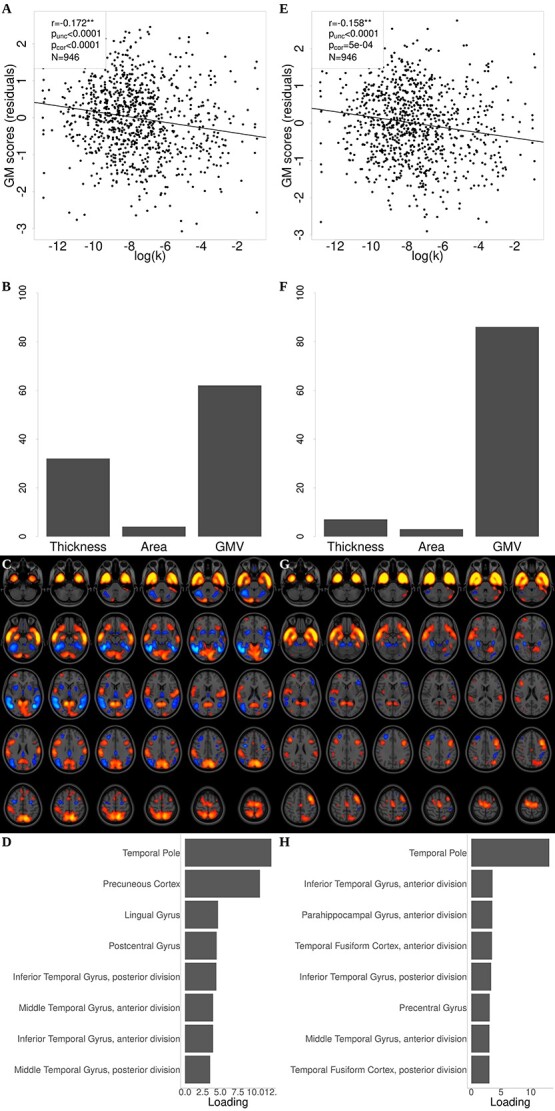
Structural component associated with DD. A) For one of the 50 gray matter components, the association between its scores and discount rates was significant. B) Relative weight of each modality to this component. C) Spatial map for the loadings corresponding with the modality with highest weight (GMV). Red (blue) color in the spatial maps denotes positive (negative) loadings. D) Loadings for the regions with highest loadings. The loadings for this component were highest in bilateral temporal areas. The panels on the right column (E–H) are equivalent to (A–D), but for the significant component found when the ICA decomposition was performed with 100 components as opposed to 50. The results for both decompositions were thus very similar. See Section 2 for details about how the components and respective scores were computed. Larger *k* (or log(*k*)) values denote a greater discounting of future rewards and reflect more impatient behavior. The *y*-axis in the scatterplots shows residuals after correcting for linear and quadratic age terms, sex, education, and income. ^**^ Statistically significant with FWER control for multiple tests (*P*_corr_ < 0.05, controlling for 50 or 100 components respectively).

Cortical thickness, surface area and GMV maps were decomposed jointly with linked-ICA in 50 components, each of them defining a spatial map reflecting the regions where gray matter structure covaried strongly across participants and individual scores indicating the magnitude of the component’s contribution. The scores of one of the gray matter components were significantly negatively associated with DD (Fig. 2A, *r* = −0.172, *P*_corr_ < 1e−4, corrected for the 50 components tested, *n* = 946). Out of the 3 gray matter modalities, this component weighted most heavily (62%) on GMV (Fig. 2B), and its largest loadings were in the temporal pole and precuneus (Fig. 2C and D). Because ICA suffers from sign indeterminacy, the scores of a component have to be interpreted with respect to its loadings (whose anatomical distribution is represented by the corresponding spatial map), i.e. a negative association with the scores of one component reflects a (partial) negative correlation with gray matter structure measurements in the areas where the values of its loadings are positive. These associations were corrected for linear and quadratic age terms, sex, education, and income. When decomposing the data in 100 components, similarly only one component was significantly associated with DD, showing a pattern very similar to the one retrieved with the 50-component decomposition (Fig. 2E–G, *r* = −0.158, *P*_corr_ = 5e−4, corrected for the 100 components tested, *n* = 946). The univariate voxel-wise analysis also revealed a negative significant association between GMV and DD in bilateral temporal gray matter regions after controlling for linear and quadratic age terms, sex, education, and income (Supplementary Fig. 2). These regions matched closely the spatial pattern for the gray matter component identified (Fig. 2C and G). There was no cortical region where the association between DD and cortical thickness or area was significant after FWER correction for multiple tests in the surface-based vertex-wise analysis for these measures.

To ensure that the association with gray matter scores was specific to DD and not merely reflecting general cognitive function, we repeated these analyses adding a measure of general intelligence to the set of covariates of no interest. The same component was still the only one that was significantly associated with DD (50-component decomposition: *r* = −0.145, *P*_corr_ = 0.001; 100-component decomposition: *r* = −0.138, *P*_corr_ = 0.123), and the result of the univariate voxel-wise analysis remained largely unchanged (Supplementary Fig. 2).

### Relationship between DD and white matter

We decomposed the FA and MD data jointly in 50 components. None of the components were associated with discount rate after controlling for multiple tests and covarying for linear and quadratic age terms, sex, education, and income. Using 100 components or controlling additionally for general intelligence yielded similar results. Correspondingly, there was no region where the association between DD and FA or MD was significant after FWER correction for multiple tests in the voxel-wise analysis.

## Discussion

After examining behavioral and neuroimaging data from a large sample of healthy adults, our findings show that greater DD was predicted by smaller anterior temporal GMV. The size of the HCP dataset sample, larger than that from most previous studies linking DD and brain structure, granted us the power to reliably capture effects of a small magnitude. However, in contradiction with former reports, we found no evidence of a reliable association between DD and cortical volume, subcortical volumes, white matter microstructural organization, and scores in WM or EM tasks.

Greater discounting was associated with smaller GMV in anterior temporal regions. This result was robust to changing the number of linked-ICA components and to control for a measure of general intelligence. Owens et al. (2017) analyzed cortical volume from T1-weigthed scans also in the HCP dataset and reported associations between cortical volume and DD in temporal regions corresponding to the areas in the gray matter component identified by our analysis. The association with anterior temporal gray matter is expected (as we used the same dataset), but our study extends the previous one by also examining cortical thickness and surface area and GMV for subcortical regions, as well as measures of white matter structure. Beyond the fact that we have included a more thorough set of structural measures, we respected the family structure of the data in computing the associations between brain structure measures and DD to avoid biased estimates that may ensue from incorrectly assuming independence of the observations (Winkler et al. 2015). We also performed a more stringent selection of subjects, excluding those who were performing at chance, and decomposed the data in structural covariance networks (Pehlivanova et al. 2018), a method that affords greater sensitivity to detect associations with DD by virtue of averaging together (in a weighted manner) values of the structural images across brain regions and combining several modalities to produce more robust estimates. Indeed, an important advantage of using ICA to decompose imaging data in covariance networks over a voxel-wise approach for analysis of structural data is that it yields scores that should be less noisy than voxel-based values because they are obtained as a weighted average of the signal in all voxels across the brain, implementing dimensionality reduction that may decrease the risk of type II errors, albeit at the expense of a vaguer anatomical delineation.

In contrast to the study by Owens et al. (2017), in our analysis the relationship between cortical volume and DD did not reach significance. This divergence is likely to be the product of our more stringent analysis choices (removal of subjects responding at random, nonparametric tests accounting for data family structure, stricter control for confounds) and indicates that the previously reported association between cortical volume and DD corresponds possibly to a spurious rather than a robust finding. It seems plausible that an association of DD with a measure of global cortical volume should be mediated by variables that reflect more general aspects of cognition than DD.

In spite of the large sample size of the HCP dataset, we found no evidence of a significant association between white matter structure and DD. Previous studies showing tractographic reconstructions in smaller samples have reported negative associations between DD and structural connectivity strength measures (e.g. number of streamlines) in specific white matter tracts to the PFC (van den Bos et al. 2015; Hänggi et al. 2016; Hampton et al. 2017). In contrast with those studies investigating connectivity strength, we analyzed FA maps processed with TBSS, a technique that enables a more wide-ranging analysis of white matter regions and is more suitable for exploratory analysis of large-scale datasets, due to the computational and labor costs associated with performing comprehensive tractographic mapping on a large number of subjects. To our knowledge, the sample in our analysis is substantially larger than in previous studies linking white matter to discounting. We note, however, that a recent study employing TBSS on 302 older subjects without dementia (Han et al. 2018) (mean age = 81.38 years, SD = 7.57 years, 75.5% female) reported widespread negative associations between temporal discounting and FA in bilateral frontal, fronto-striatal, and temporoparietal tracts. The disagreement with our results may respond to demographic differences in the samples. In view of the extensive nature of their associations, their findings may be reflecting aging-related degenerative mechanisms, as opposed to the more confined pattern that would be expected for a specific measure as DD. Besides, Han et al. did not control for income level. Indeed, when only adjusting for age and sex in our analysis, we identified further gray and white matter components that had scores significantly correlated with discount rates (see Supplementary Material). While large samples are capable of detecting smaller effects, there is also a higher risk to detect associations merely produced by confounding variables, which makes it key to adjust for the appropriate covariates.

Recent work (Marek et al. 2022) has emphasized the importance of using large samples in brain-wide brain–behavior association studies and shown that typical sample sizes for these studies should lead to irreproducible effects and inflated effect sizes. Assuming that the true effect is about 0.15 (comparable to the effect size for the gray matter component, we identified and compatible with the results from Marek et al. 2022), over 250 subjects would be needed to achieve a power of 80%. Most previous studies in the area of DD have thus been underpowered, which explains the literature inconsistencies mentioned in Section 1. Please note that this conclusion applies to brain–behavior associations only and not to functional brain mapping studies on DD, which should require smaller sample sizes to detect true effects, and therefore, there is no contradiction between our results and findings from those studies.

We could did not find an association between a preference for immediate rewards and any of the WM tests (LSWMT and 2-back). Similarly, there was no association of DD with either verbal or nonverbal EM in the HCP data. These results are in line with a recent study (Yeh et al. 2021), which we complement by including the 2-back task and a more strict statistical treatment, as for the analysis of brain structural data. Although Shamosh et al. (2008) reported a link between lower WM and a preference for immediate rewards, this correlation was not specific of WM, as in their data this ability did not explain variance in DD beyond that explained by general intelligence. When correcting the associations for age and sex only, DD was significantly negatively correlated with LSWMT, 2-back, and verbal EM scores (see Supplementary Material), but this correlation was no longer significant when adjusting for socioeconomic status variables (which should already account partly for general cognitive function). Thus, our considerations above regarding spurious associations due to nuisance variables and irreproducible findings when using small samples also apply to these analyses.

A limitation of our study is that our analyses were not preregistered, and therefore, the reported associations cannot be regarded as confirmatory. Nevertheless, we believe that an important contribution of these results is in providing more precise estimates of effect size for relationships between cognitive measures, structural imaging, and intertemporal choices due to the considerably larger sample size with respect to previous studies. Another limitation is the low reliability of discount rates. Task parameters have been criticized for their low test–retest reliability (Enkavi et al. 2019) and, in the related domain of risk preferences, for lacking consistency across experimental paradigms (Pedroni et al. 2017). These reasons may partly explain the paucity of associations with brain structure we found and the low reproducibility of findings across studies. A crucial future avenue should then be to design novel paradigms to measure DD with improved reliability. A possible way forward may lie in deriving summary scores combining several task parameters to achieve higher reliability and using multivariate methods to increase the chances of finding brain–behavior associations (Moutoussis et al. 2021).

Considering the importance of DD in the study of psychopathology, it is fundamental to robustly determine its underlying neurobiological scaffolding. By leveraging a large neuroimaging dataset, the present study helps reconcile disparities in the literature on gray and white matter correlates of DD. This behavioral trait was negatively associated with GMV in anterior temporal regions and, importantly, the structural effect identified was small, such that it would be unlikely to be detected in samples with a size comparable to that found in many related studies. All in all, associations with cognitive abilities and brain structure may be feebler than previous reports suggest. Our results call for the development of more robust measures of DD and the implementation of neuroimaging studies of larger sample size than what has been common in the field as well as appropriate control for possible confounders.

## Supplementary Material

SupplementaryMaterial_bhac164
