# Supplementary material for: Investigating associations of delay discounting with brain structure, working memory, and episodic memory
Source: Cereb Cortex. 2022 Apr 30;33(5):1669–78. doi: 10.1093/cercor/bhac164 (PMC9977379; doi:10.1093/cercor/bhac164)
Supplement: SupplementaryMaterial_bhac164 [file supplementarymaterial_bhac164.docx]

# Supplementary Methods

## Delay discounting task

This task has been described elsewhere (Barch et al. 2013), but it is summarized here for completeness. The task had a total of 60 trials and two phases, corresponding to small and large delayed monetary amounts. In the first half of the trials, the delayed amount offered was 200 USD, whereas in the second half the amount was 40 000 USD. Each phase consisted of six groups of five trials. Within each of these groups of trials the delay for the future reward was constant, and immediate rewards (a fraction of the delayed rewards) were adjusted on a trial-by-trial basis contingent on participants' responses to hone in on indifference points (Estle et al. 2006). Delays for the future reward varied across the six groups of trials, between 1 month and 10 years.

### Delay discounting model

We modelled participants’ subjective values of the rewards presented with a hyperbolic discounting function (Mazur 1987), with discount rate $k$. $R_{St}$ and $R_{Lt}$ are, respectively, the sooner and later rewards on trial *t*, presented with corresponding delays $d_{St}$ and $d_{Lt}$ (in days), and subjective values $V_{St}$ and $V_{Lt}$. Sooner delays were immediate (i.e. $d_{St}=0$), and therefore:

$$V_{St}=\frac{R_{St}}{1+kd_{St}}=R_{St}$$

$$V_{Lt}=\frac{R_{Lt}}{1+kd_{Lt}}$$

Subjects’ choices $c_{t}$ (1 = later, 0 = sooner) followed a Bernoulli distribution governed by a parameter obtained via sigmoidal transformation of the difference between values $\Delta V=R_{Lt}-R_{St}$.

$$c_{t}\sim Bernoulli(\frac{1}{1+e^{-\beta\Delta V}})$$

$\beta$ is an inverse temperature parameter controlling how deterministically choices follow the subjective difference in the value of the presented options.

To infer the posterior distributions of the free parameters of these models, $k$ and $\beta$, we used No-U-Turn Monte Carlo sampling (NUTS), as implemented in the rstan package 2. 17.3 (<http://mc-stan.org/>) for R. We employed 4 chains with 3000 warmup and 1000 post-warmup iterations. The potential scale reduction factor $\hat{R}$ was below 1.01 for all parameters, indicating effective convergence of the chains. We used the hierarchical implementation from the hBayesDM package (Ahn et al. 2017). The posterior mean estimate of $k$ for each subject was log-transformed the estimates to render the values normally distributed for subsequent analyses.

### Small vs large magnitude trials

Besides fitting the model to the full set of trials completed by the subjects, we also estimated the parameters fitting the model separately to small (delayed amount 200 USD) or large (delayed amount 40000 USD) magnitude trials only. There was a high correlation between $log(k)$ obtained from small vs large magnitude trials (r = 0.74, p < 1e-15, n = 1018, see Supplementary Figure 1). For small magnitude trials vs the full set, the correlation was equal to 0.74 (p < 1e-15, n= 1018) and for large magnitude trials, 0.99 (p < 1e-15, n = 1018). This indicates that discount rates derived from the full set of trials reflected mainly discount rates for large magnitude trials, yet they were highly correlated with small magnitude discount rates.

### Parameter-recovery control analysis

To check the if the estimated parameters were meaningful, we sampled 500 subjects from the dataset without replacement and for each of them simulated task responses with their estimated $k$ and $\beta$ parameters. We then fitted the above model to the simulated responses to estimate again these parameters. The correlation between true (i. e. values used to simulate responses) and recovered parameters was r = 0.64 (p < 1e-10, n = 500) for $log(k)$ and r = 0.80 (p < 1-e-10, n = 500) for $log(\beta)$ when using the full set of trials. Therefore, although retrieval was not perfect and was more modest for discount rates, both parameters could be recovered to a large extent.

The parameter-recovery control analyses applied separately to small and large magnitude trials, showed that the correlation between true and recovered parameters was considerably lower when fitting only small (r = 0.51, p < 1e -15, n = 500 for $log(k)$; r = 0.39, p < 1e-15, n = 500 for $log(\beta)$) or large magnitude trials (r = 0.58, p < 1e -15, n = 500 for $log(k)$; r = 0.72, p < 1e-15, n = 500 for $log(\beta)$). In view of these results and the high correlation found between $log(k)$ values computed from small vs large magnitude trials, we opted to use full set of trials when estimating discount rates in the remainder of analyses.

## Working memory scores

Participants performed one WM task inside the scanner and one outside the scanner. From the task inside the scanner we only analyzed task responses.

Outside the scanner, subjects were required to complete the NIH Toolbox List Sorting Working Memory Test (LSWMT; <http://www.nihtoolbox.org>) (Tulsky et al. 2014). Pictures of different foods and animals were displayed with both a sound clip and written text that named the item. The examinee was required to remember each stimulus in a series, mentally reorder them from smallest to largest, and recite the names of the stimuli in that order. The task had two different conditions: 1-List and 2-List. In the 1-List condition, participants were required to order a series of objects (either food or animals) in size order from smallest to largest. In the 2-List condition, participants were presented both food and animals and were asked to report the food in size order, followed by the animals in size order. Subjects had two practice items in each condition. The score was the standardized sum of total correct responses across both lists.

Inside the scanner, subjects performed two runs of an n-back task (Barch et al. 2013) (we only analyzed tasks responses, and not the fMRI data). Each run was divided in blocks of trials that consisted of pictures of either faces, places, tools, or body parts (trials were divided into these four stimulus categories for purposes that are not relevant for our analysis). Within each run, 1/2 of the blocks used a 2-back WM task (the subject had to respond ‘target’ whenever the current stimulus was the same as the one presented two trials before) and 1/2 used a 0-back WM task (in which a target cue was presented at the start of each block, and the person had to respond ‘target’ to any presentation of that stimulus during the block). A 2.5 s cue indicated the task type (and the stimulus target for 0-back) at the start of the block. Each of the two runs contained 8 task blocks (10 trials of 2.5 s each, for 25 s) and 4 fixation blocks (15 s each). On each trial, the stimulus was presented for 2 s, followed by a 500 ms ITI. Each block contained 10 trials, of which 2 were targets, and 2–3 were non-target lures (e.g., repeated items in the wrong n-back position, either 1-back or 3-back). The score considered was the accuracy (correct answers/total answers) in the 2-back task, including all stimulus categories.

## Episodic memory scores

Verbal EM was measured using the Penn Word Memory Test (PWMT) (Gur et al. 2001), which presents 20 target words that participants are asked to try to remember. After the encoding phase, targets are mixed with 20 distractors matched on memory-related characteristics (frequency, length, concreteness, and low imageability), and participants are asked to recognize the targets. They are then shown 40 words (the 20 previously presented words and 20 new words matched on memory-related characteristics). The score reflects the number of correctly recognized targets and correctly rejected distractors. Non-verbal EM was assessed with the NIH Toolbox Picture Sequence Memory Test (PSMT; <http://www.nihtoolbox.org>) (Tulsky et al. 2014). This task requires subjects to recall increasingly longer series of illustrated objects and activities that are presented in a particular order on the computer screen. Participants are asked to recall the sequence of pictures that is demonstrated over two learning trials with sequence length varying from 6-18 pictures. Participants are given a point for each adjacent pair of pictures they correctly place, up to the maximum value for the sequence, which is one less than the sequence length (if there are 18 pictures in the sequence, the maximum score is 17, because that is the number of adjacent pairs of pictures that exist). The score for this task is the total number of points received.

# Magnetic resonance imaging

### Data acquisition

The parameters of the HCP acquisition have been described elsewhere (Glasser et al. 2013; Sotiropoulos et al. 2013; Uǧurbil et al. 2013). In brief, scans were collected with a customized Siemens Magnetom Connectome 3T scanner (Siemens, Erlangen, Germany) a 32-channel head coil. Two separate averages of the T1w image were acquired using the 3D MPRAGE sequence with 0.7x0.7x0.7 mm^3^ isotropic resolution (FOV = 224 mm, matrix = 320, 256 sagittal slices in a single slab, TR = 2400 ms, TE = 2.14 ms, TI = 1000 ms, flip angle = 8°, phase encoding factor GRAPPA = 2).

Diffusion data were collected using a single-shot, single refocusing spin-echo, echo-planar imaging sequence with 1.25 mm isotropic spatial resolution (TR = 5520 ms, TE =89.5 ms, FOV=21 × 18 cm). Three gradient tables of 90 diffusion-weighted directions and six b=0 images each were collected with right-to-left and left-to-right phase encoding polarities for each of the three diffusion weightings (b = 1000, 2000, and 3000 s/mm^2^).

### MRI data preprocessing

### T1-weighted images

We processed the T1-weighted images with FSL-VBM (<http://fsl.fmrib.ox.ac.uk/fsl/fslwiki/FSLVBM>) (Douaud et al. 2007). The structural images were brain-extracted and gray matter-segmented before being registered to MNI152 standard space using non-linear registration (Andersson et al. 2007). The resulting images were averaged and flipped along the x-axis to create a left-right symmetric, study-specific gray matter template. Native gray matter images were then non-linearly registered to this study-specific template and multiplied by the Jacobian of the warp field to compensate for local expansion/contraction due to the non-linear component of the spatial transformation. The modulated gray matter images were then smoothed with an isotropic Gaussian kernel with full-width at half-maximum (FWHM) of 8 mm to produce gray matter volume maps (GMV).

Segmentation of subcortical structures (accumbens, amygdala, caudate, hippocampus, pallidum, putamen and thalamus), reconstruction of the cortical surface and estimation of its total volume were performed with Freesurfer 5.3 (Fischl and Dale 2000). The pipeline for the HCP data, given its higher resolution and particular characteristics, involved several further steps that have been described in detail elsewhere (Glasser et al. 2013). Individual maps of thickness and area were warped onto the 164k_fs_LR mesh ([http://brainvis.wustl.edu/wiki/index.php//Caret:Atlases/Conte69_Atlas](http://brainvis.wustl.edu/wiki/index.php/Caret:Atlases/Conte69_Atlas)) and smoothed with an isotropic 2D Gaussian kernel with FWHM of 10 mm. These operations were performed with Freesurfer commands and Connectome Workbench (<https://www.humanconnectome.org/software/connectome-workbench>).

Estimating surface-based cortical thickness requires identifying the cortical boundaries between cerebro-spinal fluid and gray matter (the so-called pial surface), and between gray and white matter (the so-called white surface) by reconstructing a computerized triangular mesh based on intensity differences in T1-weighted images (Fischl and Dale 2000). Cortical thickness at each surface vertex is then obtained as the distance between the pial and white surfaces along their perpendicular direction. Surface area is estimated as one-third of the sum of the areas of all faces of the white surface that have that vertex in common (Winkler et al. 2012). By registering and resampling subject-specific meshes to a common grid, it is possible to compare local surface area across subjects at different cortical regions (Winkler et al. 2018). Notably, cortical thickness and area measure biologically distinct processes (Winkler et al. 2018). In particular, they are genetically uncorrelated (Winkler et al. 2010), under the influence of separate genetic factors (Chen et al. 2015) and evolve differently over the lifespan (Fjell et al. 2015).

In VBM, T1-weighted images are initially probabilistically segmented in cerebro-spinal fluid and gray and white matter. The value at each voxel in the resulting tissue segments approximates the proportion of the corresponding tissue in that voxel, and is scaled by the amount of regional deformation necessary to warp a subject’s image to a common template, so that the resulting value at each voxel can be regarded as a measure of local GMV (Ashburner and Friston 2000, 2005). An important advantage of VBM estimates of GMV with respect to cortical measures, is that they can also be measured within subcortical structures (allowing, for example, to study cortico-subcortical structural covariance networks as opposed to only cortico-cortical ones). Previous investigations have combined surface-based and VBM measures, recognizing these measures as complementary techniques to study gray matter properties (Voets et al. 2008; Groves et al. 2012; Douaud et al. 2014; Kong et al. 2015).

### Diffusion Imaging

Diffusion data were processed with FSL’s FMRIB's Diffusion Toolbox (FDT; https://fsl.fmrib.ox.ac.uk/fsl/fslwiki/FDT). We used already-processed data which are available for download and have been obtained using the steps described in (Glasser et al. 2013). Finally, we computed fractional anisotropy (FA) and mean diffusivity (MD) maps by fitting a diffusion tensor to the data. The FA/MD maps were processed with the tract-based spatial statistics (TBSS) pipeline (Smith et al. 2006) with default parameters. In brief, individual FA maps were non-linearly normalized to the FMRIB58_FA template with FNIRT (Andersson et al. 2007). Normalized FA images were averaged and the mean FA image thinned to create a mean FA skeleton, a map representing the central region of all tracts generally common to the study participants. Each subject's aligned FA image was then projected onto the mean skeleton, resulting in individual FA skeleton images. The same transformations were then applied to the MD maps to produce individual MD skeleton images.

### Independent Component Analysis

We used FSL’s implementation of the algorithm (<http://fsl.fmrib.ox.ac.uk/fsl/fslwiki/FLICA>, (Groves et al. 2011)), which uses variational Bayesian inference to estimate the sources of the signal, running on MATLAB R2017b. We produced a decomposition with 50 components, which rendered interpretable results. Although examination of the scree plot of an initial principal-component decomposition suggested that the data could be decomposed further, this would have led to impractical computation times and resource requirements along with the need to control for a larger number of tests. A previous study performed on a similar dataset (Groves et al. 2012) showed that when using 100 components instead of 50, most components remained stable and many of the new dimensions were filled with additional components that would otherwise have been treated as noise. For each set of imaging measures (gray or white matter structural measurements), the decomposition yielded a score $S_{ij}$ for each subject $i$ and component $j$, reflecting how strongly that particular component is represented in that subject, and modality weights $W_{jm}$ indicating the relative contribution to component $j$ of each modality $m$ (cortical thickness, surface area or GMV for gray matter components and FA or MD for white matter components). The scores can be arranged in matrices $\bar{\boldsymbol{S}}$ (subjects x components). Each component is accompanied by a map encoding spatial variation of its loadings across the brain. In order to interpret anatomically these spatial maps, we computed the probability that the spatial map was a member of the different labelled regions within predefined atlases, weighting this probability by the value of the spatial map to obtain a loading for each atlas region. For volume-based maps we computed these loadings with FSL’s Atlasquery tool, using the Harvard-Oxford cortical atlas for the GMV maps and the JHU white matter tractography atlas for the FA/MD maps. For cortical thickness we used the mri_segstats command from the Freesurfer package and the Desikan-Killiany cortical atlas (Desikan et al. 2006).

# Supplementary Results

### Relationship between delay discounting and gray matter, controlling only for age and sex

The scores of three of the components of the 50-component ICA decomposition of cortical thickness, surface of and GMV and surface area were correlated with DD when controlling for linear and quadratic age terms and sex. The scores of one of the gray matter components (GM1) were positively associated with DD (r = 0.119, p = 0.024, corrected for the 50 components tested, n = 946). Out of the three gray matter modalities, this component weighted most heavily (83%) on cortical thickness, and loaded on parietal and frontal regions bilaterally, with the largest weight on superior parietal cortex. A second gray matter component (GM2) was strongly and negatively associated with DD (r = -0.197, p < 1e-4, corrected for the 50 components tested, n = 946). The largest modality contribution for this component (62 %) was from GMV, and the largest loadings in the temporal pole and precuneus (this component corresponds to the one reported in the main text. The effect on GM1 was no longer significant after adjusting for education and income in addition to age and sex, and therefore in the main text we only discuss the association which was robust, namely with GM2.

### Relationship between delay discounting and white matter, controlling only for age and sex

The scores of one of the components of the 50-component ICA decomposition of FA and MD were positively associated with DD, controlling for linear and quadratic age terms and sex (r = 0.126, p = 0.017 corrected for the 50 components tested, n = 905). This component had its largest relative weight (69 %) on FA measurements and encompassed frontal white matter (loading most highly on forceps minor, anterior thalamic radiation, inferior fronto-occipital fasciculus). However, as explained in the main text, this effect was no longer significant after adjusting for education and income in addition to age and sex.

### Relationship between delay discounting and working/episodic memory, controlling only for age and sex

After controlling for linear and quadratic age terms and sex, discount rate was significantly associated with LSWMT scores (r = -0.076, p = 0.048, n = 1018) and marginally significantly associated with 2-back scores (r = -0.076, p = 0.055, n = 923). Thus, subjects with worse WM performance discounted future rewards more. Subjects with worse verbal EM scores (PWMT) discounted future rewards more (r = -0.091, p = 0.013, n = 1018), even after controlling for performance in the WM tasks (r = -0.076, p = 0.026, n = 923). The association with non-verbal EM scores (PSMT) was not significant (r = -0.019, p = 0.695, n = 1017). As explained in the main text, these effects were no longer significant after adjusting for education and income in addition to age and sex.

# Supplementary Figures


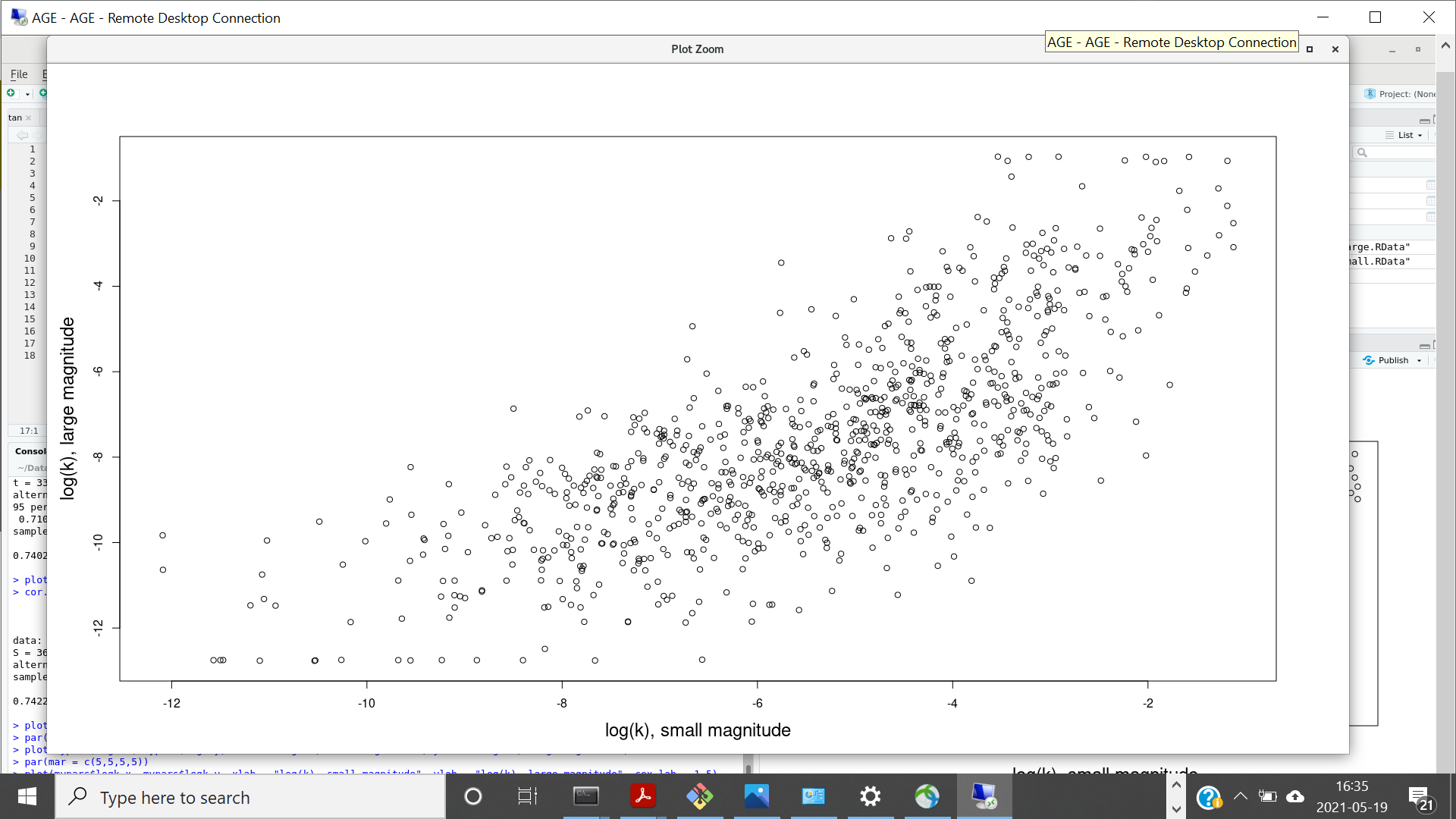


**Supplementary Figure 1. Comparison of log(k) values computed using small vs large magnitude trials.** The correlation was (r = 0.74), p < 1e-15, n = 1018).


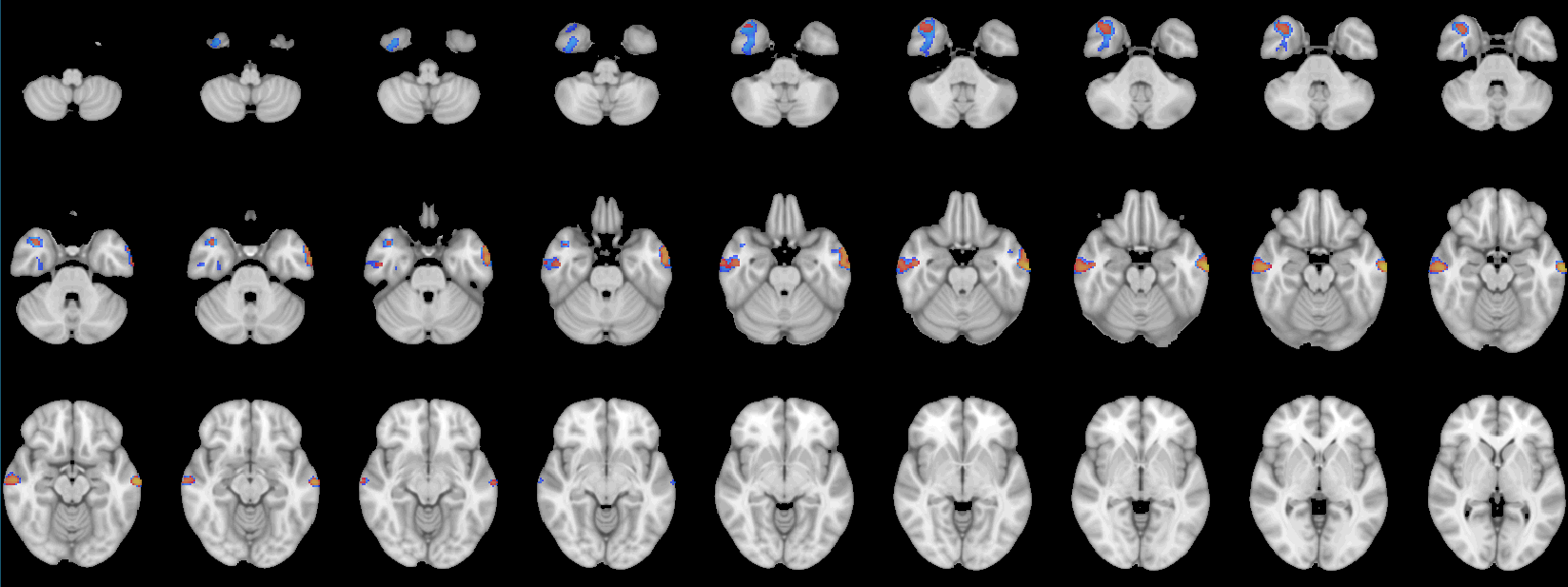


**Supplementary Figure 2. Voxel-wise analysis of GMV.** In blue, brain regions where GMV was significantly associated with DD after controlling for linear and quadratic age terms, sex, education and income (FWER corrected, p < 0.05). In red, additionally controlling for general intelligence.

# Supplementary References

Ahn W-Y, Haines N, Zhang L. 2017. Revealing neuro-computational mechanisms of reinforcement learning and decision-making with the hBayesDM package. Comput Psychiatry. 1:24–57.

Andersson JLR, Jenkinson M, Smith S. 2007. Non-linear registration aka Spatial normalisation FMRIB Technial Report TR07JA2.

Barch DM, Burgess GC, Harms MP, Petersen SE, Schlaggar BL, Corbetta M, Glasser MF, Curtiss S, Dixit S, Feldt C, Nolan D, Bryant E, Hartley T, Footer O, Bjork JM, Poldrack R, Smith S, Johansen-Berg H, Snyder AZ, Van Essen DC. 2013. Function in the human connectome: Task-fMRI and individual differences in behavior. Neuroimage. 80:169–189.

Desikan RS, Se F, Fischl B, Quinn BT, Dickerson BC, Blacker D, Buckner RL, Dale AM, Maguire RP, Hyman BT, Albert MS, Killiany RJ. 2006. An automated labeling system for subdividing the human cerebral cortex on MRI scans into gyral based regions of interest. 31:968–980.

Douaud G, Smith S, Jenkinson M, Behrens T, Johansen-Berg H, Vickers J, James S, Voets N, Watkins K, Matthews PM, James A. 2007. Anatomically related grey and white matter abnormalities in adolescent-onset schizophrenia. Brain. 130:2375–2386.

Estle SJ, Green L, Myerson J, Holt DD. 2006. Differential effects of amount on temporal and probability discounting of gains and losses. Mem Cognit. 914–928.

Fischl B, Dale AM. 2000. Measuring the thickness of the human cerebral cortex from magnetic resonance images. Proc Natl Acad Sci U S A. 97:11050–11055.

Glasser MF, Sotiropoulos SN, Wilson JA, Coalson TS, Fischl B, Andersson JL, Xu J, Jbabdi S, Webster M, Polimeni JR, Van Essen DC, Jenkinson M. 2013. The minimal preprocessing pipelines for the Human Connectome Project. Neuroimage. 80:105–124.

Groves AR, Beckmann CF, Smith SM, Woolrich MW. 2011. Linked independent component analysis for multimodal data fusion. Neuroimage. 54:2198–2217.

Groves AR, Smith SM, Fjell AM, Tamnes CK, Walhovd KB, Douaud G, Woolrich MW, Westlye LT. 2012. Benefits of multi-modal fusion analysis on a large-scale dataset: Life-span patterns of inter-subject variability in cortical morphometry and white matter microstructure. Neuroimage. 63:365–380.

Gur R, Ragland JD, Moberg PJ, Siegel SJ. 2001. Computerized Neurocognitive Scanning : I . Methodology and Validation in Healthy People. Neuropsychopharmacology. 25:766–776.

Mazur JE. 1987. An adjusting procedure for studying delayed reinforcement. In: Commons ML,, Mazur JE,, Nevin JA,, Rachlin H, editors. Quantitative analyses of behavior: Vol. 5. The effect of delay and of intervening events on reinforcement value. Hillsdale, NJ: Lawrence Erlbaum Associates. p. 55–73.

Smith SM, Jenkinson M, Johansen-Berg H, Rueckert D, Nichols TE, Mackay CE, Watkins KE, Ciccarelli O, Cader MZ, Matthews PM, Behrens TEJ. 2006. Tract-based spatial statistics: Voxelwise analysis of multi-subject diffusion data. Neuroimage. 31:1487–1505.

Sotiropoulos SN, Jbabdi S, Xu J, Andersson JL, Moeller S, Auerbach EJ, Glasser MF, Hernandez M, Sapiro G, Jenkinson M, Feinberg DA, Yacoub E, Lenglet C, Van Essen DC, Ugurbil K, Behrens TEJ. 2013. Advances in diffusion MRI acquisition and processing in the Human Connectome Project. Neuroimage. 80:125–143.

Tulsky DS, Carlozzi N, Chiaravalloti ND, Beaumont JL, Kisala PA, Mungas D, Conway K, Gershon R. 2014. NIH Toolbox Cognition Battery (NIHTB-CB): The List Sorting Test to Measure Working Memory. J Int Neuropsychol Soc. 20:599–610.

Uǧurbil K, Xu J, Auerbach EJ, Moeller S, Vu AT, Duarte-Carvajalino JM, Lenglet C, Wu X, Schmitter S, Van de Moortele PF, Strupp J, Sapiro G, De Martino F, Wang D, Harel N, Garwood M, Chen L, Feinberg DA, Smith SM, Miller KL, Sotiropoulos SN, Jbabdi S, Andersson JLR, Behrens TEJ, Glasser MF, Van Essen DC, Yacoub E. 2013. Pushing spatial and temporal resolution for functional and diffusion MRI in the Human Connectome Project. Neuroimage. 80:80–104.
